# Turning the needle into the haystack: Culture-independent amplification of complex microbial genomes directly from their native environment

**DOI:** 10.1371/journal.ppat.1012418

**Published:** 2024-09-12

**Authors:** Olivia A. Pilling, Sesh A. Sundararaman, Dustin Brisson, Daniel P. Beiting

**Affiliations:** 1 Department of Pathobiology, School of Veterinary Medicine, University of Pennsylvania, Philadelphia, Pennsylvania, United States of America; 2 Department of Pediatrics, Children’s Hospital of Philadelphia, The Perelman School of Medicine, University of Pennsylvania, Philadelphia, Pennsylvania, United States of America; 3 Department of Biology, School of Arts & Sciences, University of Pennsylvania, Pennsylvania, United States of America; Joan and Sanford I Weill Medical College of Cornell University, UNITED STATES OF AMERICA

## Abstract

High-throughput sequencing (HTS) has revolutionized microbiology, but many microbes exist at low abundance in their natural environment and/or are difficult, if not impossible, to culture in the laboratory. This makes it challenging to use HTS to study the genomes of many important microbes and pathogens. In this review, we discuss the development and application of selective whole genome amplification (SWGA) to allow whole or partial genomes to be sequenced for low abundance microbes directly from complex biological samples. We highlight ways in which genomic data generated by SWGA have been used to elucidate the population dynamics of important human pathogens and monitor development of antimicrobial resistance and the emergence of potential outbreaks. We also describe the limitations of this method and propose some potential innovations that could be used to improve the quality of SWGA and lower the barriers to using this method across a wider range of infectious pathogens.

## Introduction

Pathogens and commensal microbes reside within extremely complex environments, including the mammalian gut, skin, urogenital tract, and upper airways, as well as environmental reservoirs, such as soil, water, and insect vectors. These niches require specialized adaptations, and our understanding of the nutrient requirements for microbes to survive in these habitats is extremely limited. As a result, many microbes are not readily amenable to in vitro culture in the laboratory and therefore are not experimentally tractable. One major development that opened the doors to culture-independent microbial genomics was the first use of “shotgun” metagenomic sequencing to reconstruct genomes and infer microbial function directly from high-throughput sequencing (HTS) data [1]. This work, along with computational methods developed to process the resulting complex data [2–6], represented a landmark breakthrough, demonstrating that de novo assembly and functional annotation of whole bacterial genomes was possible for organisms that had never before been cultured in the laboratory. In the 20 years since this discovery, metagenomic sequencing and de novo assembly methods have yielded rich collections of metagenome-assembled genomes (MAGs) for bacteria, shedding light on novel microbial functions and evolution [7–11]. Unfortunately, several factors limit the more widespread use of metagenomics to study microbes that cause disease. Metagenomic data are often dominated by high levels of nonmicrobial “contaminating” sequences from the host or environment. For example, stool from healthy donors may contain as little as 10% host DNA, but in patients with gastrointestinal inflammation, this can increase to over 90% [12–14]. Similarly, saliva, nasal, buccal, and vaginal samples routinely comprise as much as 90% host DNA [14]. This issue, together with the relatively high cost associated with deep sequencing, often renders metagenomic methods inefficient for studying microbial genomes that are present at low abundance in complex samples. Recent innovations in computational and sequencing methods may ameliorate these issues, but even in the complete absence of contaminating host sequences, HTS of mixed communities often yields poor coverage of larger genomes, such as those from microbial eukaryotes including helminths, protozoa, and fungi. This makes assembly of whole or partial genomes of these microbes from metagenomic data problematic [15,16].

A hallmark of many infectious diseases is the chronic persistence of pathogens at low levels in host tissues, thereby avoiding immune-mediated clearance and maximizing the chance of transmission. Many parasites like *Trypanosoma cruzi* and *Toxoplasma gondii* establish persistent, often lifelong, infections that remain at low densities for decades. Similarly, *Mycobacterium tuberculosis* and *Mycobacterium bovis*, the cause of human and bovine tuberculosis, respectively, are extremely slow growing and develop latent infections that are difficult to directly detect [17,18]. Many chronic bacterial pathogens like *Borrelia burgdorferi*, the cause of Lyme disease, also persist at extremely low levels in human or animal hosts [19]. These examples underscore the challenges associated with studying the genomes of important human and animal pathogens.

In this review, we describe the development and use of selective whole genome amplification (SWGA) to extract whole or partial microbial genomes directly from their native animal and human hosts, even when the organism is present at extremely low abundance. SWGA uses short oligonucleotide primers designed to preferentially bind a target microbial genome over one or more contaminating “background” host genomes. These primers are used as a pool in a multiplexed isothermal reaction together with a high-fidelity, highly processive *Phi*29 polymerase to preferentially amplify large segments (up to 70 to 100 kb) of a target genome [20,21]. The simplicity and low cost of SWGA makes it possible to implement in low- and middle-income countries (LMICs) that may have limited laboratory resources. This review covers nearly a decade of SWGA research and development spanning 3 bacterial species, eukaryotic microbes including the protozoa *Plasmodium* spp. and *Leishmania braziliensis*, and the helminth parasite, *Wuchereria bancrofti*. The broad use of SWGA across a diverse range of organisms highlights the impact of this method and the potential for approaches like SWGA to serve as a general toolkit for population genomics of pathogens that may otherwise be difficult to study in vitro.

## The case for SWGA

**Fig 1:**
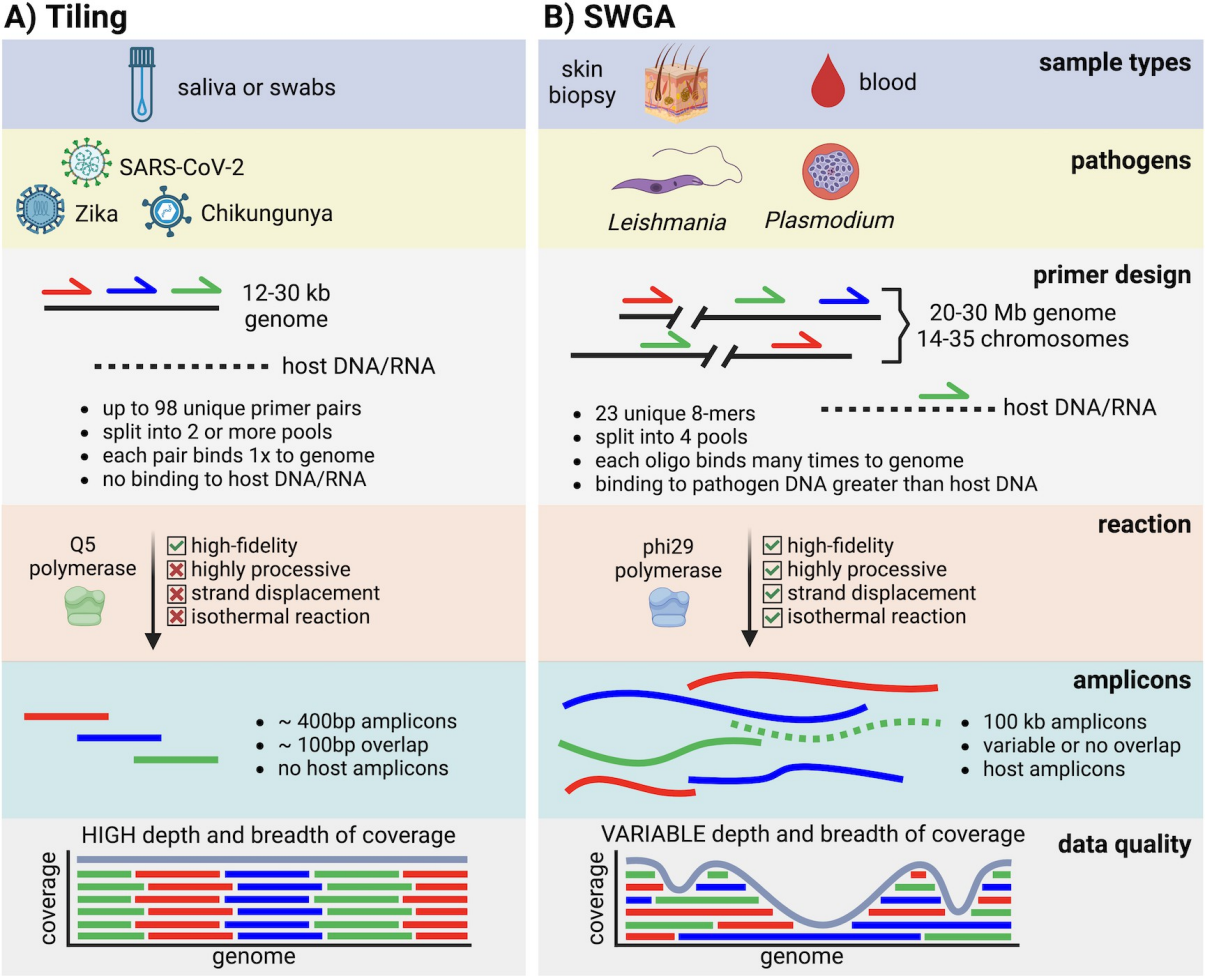
SWGA unlocks population genomics for nonviral pathogens. Schematic comparison of **(A)** tiling-based methods for viral genomic surveillance studies and **(B)** SWGA for bacterial and eukaryotic pathogens. Comparisons are made for sample type, pathogens targeted, primer design, PCR reactions, amplicons produced, and the quality of data produced by these 2 approaches. Created with Biorender.com.

To appreciate the need for, and utility of, SWGA, it is useful to first consider modern viral genomic surveillance **(Fig 1).** The small size of viral genomes, together with a high degree of sequence divergence between viruses and the human or animal host(s) they infect, means that conventional PCR primers can readily be designed [22] that will produce PCR amplicons that tile (partially overlap) across the full length of the viral genome, with little or no off-target binding to host DNA or RNA present in the original sample **(Fig 1A)**. Tiling primer sets are then combined into 2 or more pools (likely to avoid primer dimers) for PCR, and the resulting products are then purified and sequenced to generate high depth of coverage across the entire viral genome. This amplicon tiling approach proved instrumental in tracking the Zika and Chikungunya virus epidemics in South America [23], and more recently for tracking Severe Acute Respiratory Syndrome Coronavirus 2 (SARS-CoV-2) genome evolution during the Coronavirus Disease 2019 (COVID-19) pandemic [24]. However, amplicon tiling requires a relatively large number of unique primer sequences for even the smallest viral genomes. For example, 35 primer pairs are needed to amplify the approximately 11 Kb genome of Zika [23], 44 primer pairs for the approximately 12 Kb genome of Chikungunya [25], and nearly 100 primer pairs for approximately 30 Kb genome of SARS-CoV-2 [24,26,27]. The genomes of bacterial and eukaryotic pathogens are orders of magnitude larger than viruses **(Fig 1B)**, making tiling approaches impractical. Moreover, eukaryotic pathogens share a much higher degree of sequence similarity with the eukaryotic hosts they infect, resulting in primers that often exhibit off-target binding to host DNA. SWGA attempts to address these challenges by using smaller pools of roughly 5 to 12, short (8 to 12 nt) oligos—rather than pathogen-specific primer pairs—which are designed to bind with higher frequency (rather than exclusively) to the target pathogen genome compared to the host background genome **(Fig 1B)**. This distinguishes SWGA from traditional whole genome amplification (WGA), which also uses *Phi*29 polymerase but which employs random primers and therefore amplifies all genomes present in a complex sample. By combining SWGA oligos with a highly processive polymerase that exhibits strand displacement and proof-reading ability, long amplicons with low error rates can be generated for the genome of interest, thus allowing the amplification of very large pathogen genomes from complex samples that contain high levels of contaminating DNA.

## SWGA—How it started, and how it’s going

**Fig 2:**
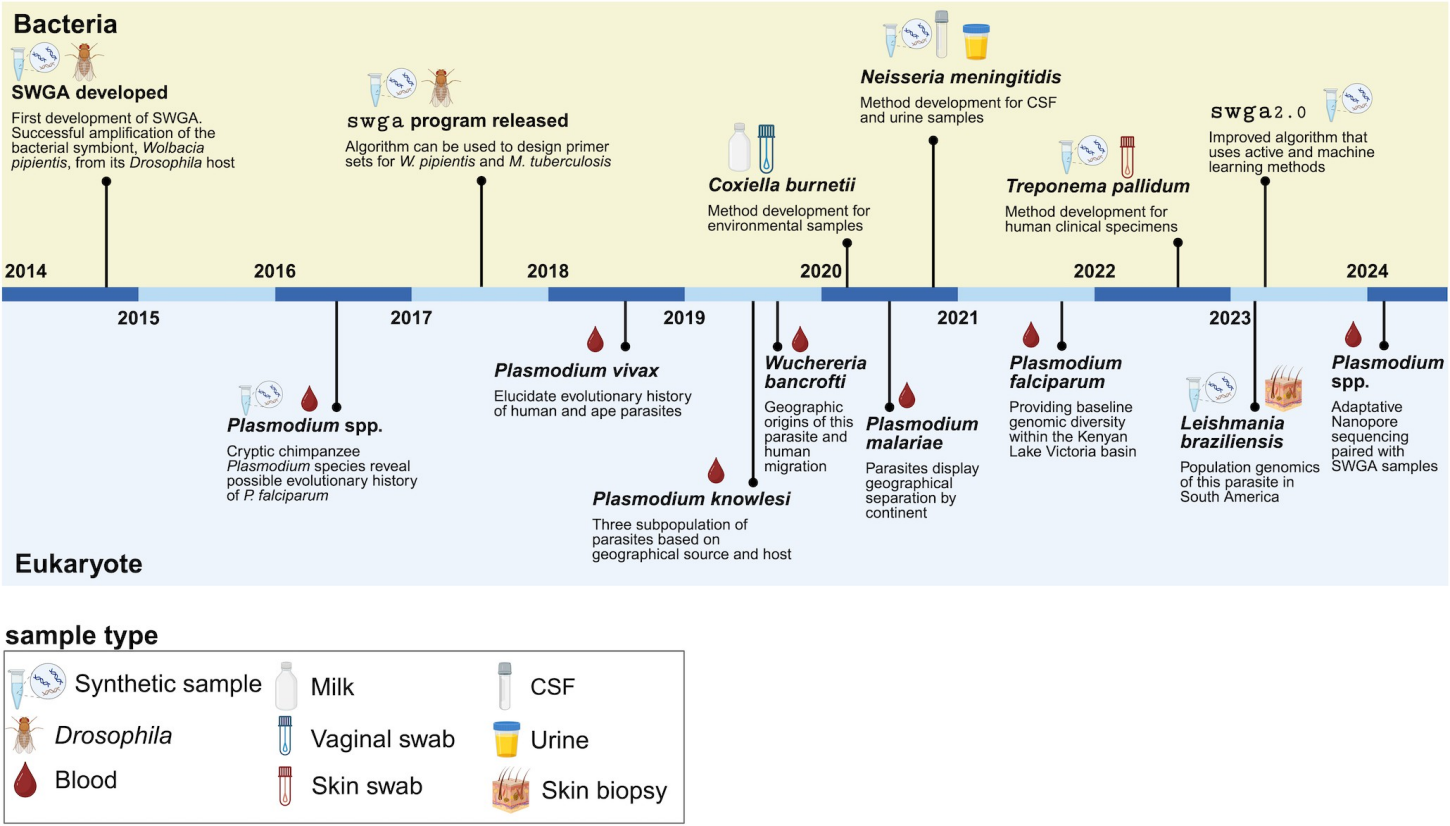
SWGA development over the past decade. Schematic showing major milestones in the development and application of SWGA across bacterial (top; yellow) and eukaryotic (bottom; blue) microbes in multiple sample types. Citations for milestones on top of timeline, from left to right are [20,21,28–30,35], and for bottom are [32,34,37,51,52,56,75,94]. Created with Biorender.com.

The first proof-of-concept use of SWGA on a complex sample involved the amplification of the bacterial symbiont, *Wolbachia pipientis*, directly from its *Drosophila* host **(Fig 2)**. Using crude extracts prepared from infected *Drosophila*, this work showed that SWGA resulted in nearly 140-fold greater amplification of *W*. *pipientis* DNA as compared to *Drosophila* DNA. This degree of enrichment yielded approximately 90% coverage of the *W*. *pipientis* genome from a modest sequencing effort of just 0.6 to 2.2 billion base pairs, 10-fold less than would have been required from unenriched samples [21]. The authors of this study later produced the first program to design of SWGA primers given any target and background (off-target) genome [20], thus opening the doors to adapt this method more broadly. Since these foundational studies, SWGA has been adapted to target several other bacterial species, including the human pathogens *Coxiella burnetii* [28], *Neisseria meningitidis* [29], and *Treponema pallidum* [30] (**Fig 2, top)**. *C*. *burnetii* is a Biosafety Level 3 (BSL3) pathogen and, as such, requires rigorous safety precautions and extensive training to handle pure cultures of this organism. SWGA enabled whole genome sequences to be generated for this pathogen directly from vaginal swabs, bypassing the need for culture [28]. Similarly, SWGA lowered the barrier to generate and study *N*. *meningitidis* genomes directly from urine and cerebrospinal fluid (CSF) [29]. Finally, Thurlow and colleagues developed SWGA for *T*. *pallidum*, the cause of syphilis, which has been difficult to study using genomics due to a paucity of in vitro culture systems [30]. The authors showed that high-quality *T*. *pallidum* genomes with ≥10× coverage across up to 98% of the genome could be generated directly from swabs of syphilis skin lesions.

**Fig 3:**
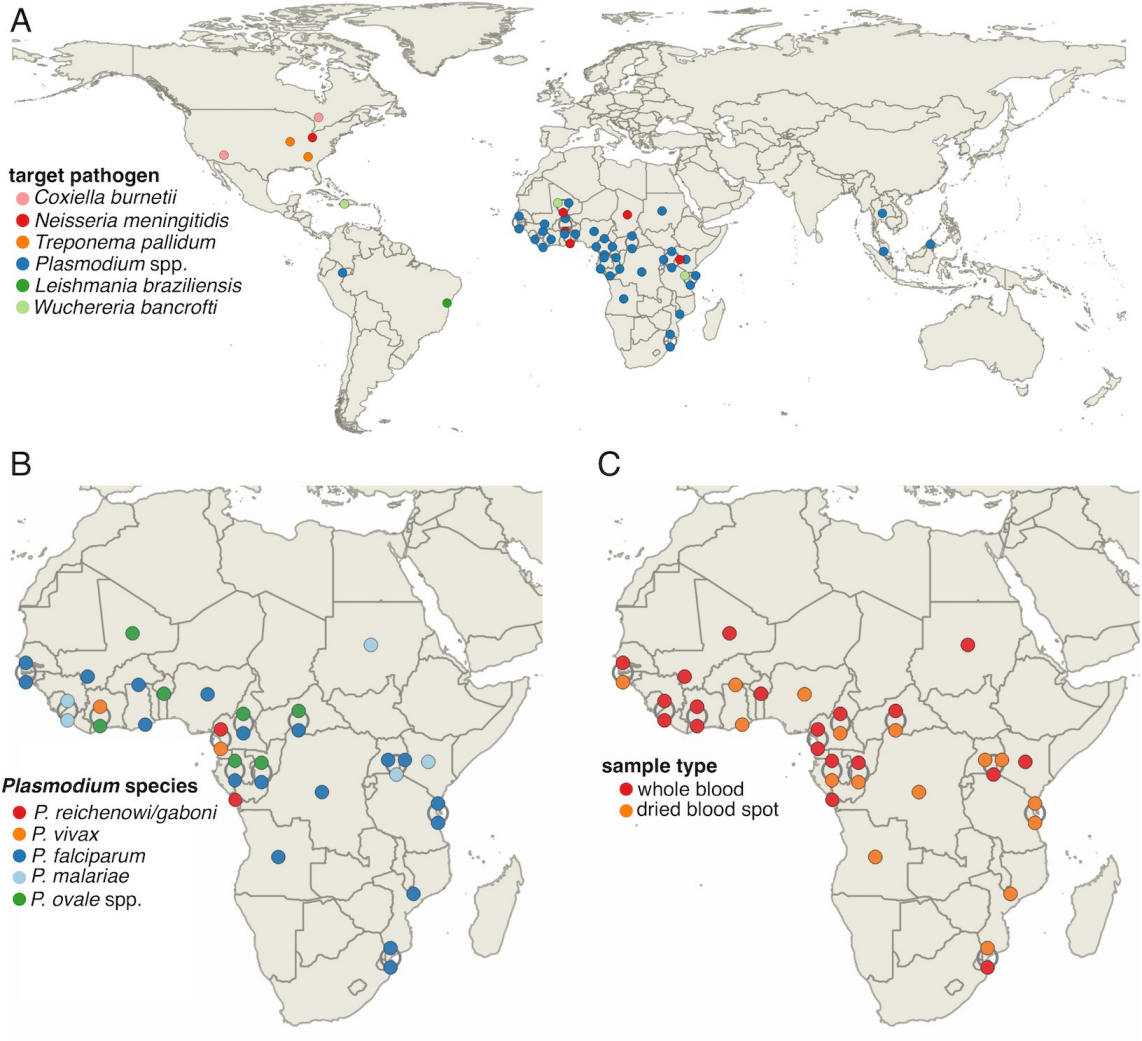
Geographic distribution of genome sequences derived from samples subjected to SWGA from the literature. **(A)** Geographic distribution colored by the pathogen targeted for study; **(B)** by *Plasmodium* species targeted; and **(C)** by sample type used for SWGA. Each point represents one or more samples from the same study for 21 or 14 published manuscripts (panels A and B/C, respectively). Data points are linked by curved lines if the points have the same GPS coordinates. Maps were created in QGIS software [95].

Although initially envisaged for bacterial organisms [21], SWGA has flourished as a tool to study eukaryotic pathogens **(Fig 2, bottom)**—most notably, *Plasmodium* species that cause malaria **(Fig 3A)**. *Plasmodium falciparum* was viewed as particularly amenable for SWGA because of the AT-rich nature of its genome (approximately 80% compared to only approximately 59% in human [31]), thus making it easier to design primers that preferentially bind to *P*. *falciparum* DNA over the background human host DNA. Remarkably, these early studies with *Plasmodium* were successful even on blood samples with parasitemia below the limit of detection by standard microscopic examination of blood smears [32]. SWGA thus enabled genomic analyses of chimpanzee and gorilla *Plasmodium* species from subclinical infections [32–34]. These species represent the closest ancestors of *P*. *falciparum* and *P*. *vivax* and revealed new insights into how these parasites evolved to infect humans. Spurred by this success, the *Plasmodium* research community has used SWGA extensively to study the population genomics of *Plasmodium* species from challenging samples with miniscule amounts of parasite DNA, including whole blood and dried blood spots (DBS) **(Fig 2, bottom)**.

The expansion of SWGA into new diseases, host species, and tissues underscored a need for a more robust algorithm for primer selection. Solid tissue specimens, such as biopsies, contain far higher levels of contaminating host DNA than fluid specimens such as blood, CSF, and urine. In addition, pathogens that are similar in AT richness to their mammalian host pose a challenge to primer design. To address these challenges, Dwivedi-Yu and colleagues recently developed open-source software that uses active and machine learning methods to improve the speed and quality of SWGA primer design [35]. As a proof-of-concept for this improved algorithm, the authors successfully developed and tested SWGA primers for *Prevotella melaninogenica*, an important pathogen in cystic fibrosis patients, but one that has similar GC content as humans and frequently eludes culture-based diagnosis and clinical epidemiology surveillance [36]. Pilling and colleagues used this improved primer design algorithm to develop SWGA primers that successfully amplified the genome of the protozoan parasite, *L*. *braziliensis*, directly from skin biopsies [37], generating ≥10× coverage across more than 80% of the 32 Mb parasite genome. This marked the first time SWGA had been applied to solid tissues, where host DNA vastly outweighs parasite genetic material.

## Population genomics powered by SWGA

Population genomics is a vital tool for revealing the evolutionary history of pathogens, shaping our view of the role people and animals play in transmission, and influencing the implementation of mitigation strategies to improve public health. SWGA has arguably advanced population genomic research for *Plasmodium* more so than for any other pathogen **(Fig 3)**. Given the low genetic diversity of *Plasmodium* species, amplicon-based sequencing methods are of little use for population level analyses. While genome-wide single nucleotide polymorphism (SNP) studies can be helpful, they require a priori knowledge of what sites are polymorphic within a population of interest. Thus, whole genome sequencing (WGS)-based methods are ideal for analyzing *Plasmodium* population-level data.

Many techniques have been developed to enrich for *Plasmodium* DNA from clinical samples prior to sequencing; however, these often require significant resources and labor at the time of collection [38–44]. DBS (small amounts of blood blotted on filter paper) have been extensively used in malaria surveillance [45]. These samples are easy to collect and transport and do not require refrigeration [46]. Unfortunately, it is impossible to separate parasite DNA from contaminating host DNA in DBS, limiting their usefulness in *Plasmodium* population genomics. SWGA overcomes these limitations, enabling WGS from DBS, even from low parasitemia samples (<0.03% parasitemia, or approximately 1,200 parasites/μl) [46,47]. Comparisons of SWGA-amplified DBS samples to venous blood assayed by traditional WGS have also showed high concordance of allele frequencies and SNP calls, indicating that genomes sequenced after SWGA are as accurate as direct sequencing and of sufficient quality for population genomic studies.

Frequent infections in malaria-endemic areas make it difficult to distinguish new infections from treatment failure due to either drug resistance or inadequate therapy. Guggisberg and colleagues overcame this using SWGA, examining treatment failure in children-administered fosmidomycin-clindamycin for *P*. *falciparum* infections [48]. The authors found that initial and recurrent infections were genetically related, but there was no evidence of selection for known drug resistance markers. These results suggest that treatment failure was most likely due to inadequate therapeutic drug concentrations and not preexisting or de novo resistance mutations. More recently, Coonahan and colleagues used SWGA to evaluate the genomic landscape of *P*. *falciparum* infections in Mozambique, identifying high levels of resistance to sulfadoxine-pyrimethamine, no evidence of continued chloroquine resistance, and multiple genomic regions with signatures of positive selection [49]. While SWGA studies have focused largely on *P*. *falciparum* (**Fig 3B**) from whole blood or DBS **(Fig 3C),** the method has also proven useful in studies of other species, including exploring transmission dynamics of *P*. *vivax*, population structure of *P*. *malariae*, and even gene flow in the zoonotic parasite *P*. *knowlesi* [50–52].

## The evolutionary history of *Plasmodium* species revealed by SWGA

Understanding the evolutionary history of pathogens can provide key insights into host–pathogen interactions and help predict the risk of future cross-species transmission. Early studies of *P*. *falciparum* and its chimpanzee relative *P*. *reichenowi* led to the hypothesis that *P*. *falciparum* had coevolved with humans over millions of years. In a landmark study, Liu and colleagues performed single genome amplification of *Plasmodium* DNA from the stool of wild-living chimpanzees, gorillas, and bonobos to show that the ancestor of *P*. *falciparum* was transmitted from gorillas into humans [53], resulting in one of the most devastating infectious diseases in human history. This finding raised key questions about the relationship of human and ape *Plasmodium* species and genetic traits that might predispose to cross-species transmission. However, the endangered status of African apes and lack of culture systems for ape parasites made comparative genomic studies challenging to carry out.

Given their protected status, the majority of blood samples from chimpanzees and gorillas in Africa are collected opportunistically (i.e., during routine health screens of apes in sanctuaries). These samples typically have extremely low levels of *Plasmodium* parasites (0.00081% to 0.14% *Plasmodium* DNA). To overcome this, Sundararaman and colleagues combined SWGA with methylation-dependent digestion (MDD) of host DNA [32]. MDD takes advantage of the differential methylation of *Plasmodium* and ape DNA to selectively degrade the latter. The addition of MDD to SWGA could improve enrichment in 2 ways. First, MDD directly degrades host DNA, thereby limiting off-target binding of SWGA primers. Second, off-target amplification of host DNA consumes deoxynucleotide triphosphates (dNTPs) and primers, which limits the total amount of DNA that can be added to a SWGA reaction while still achieving significant amplification. By decreasing off-target amplification, MDD could allow the use of more DNA and, therefore, more target *Plasmodium* genomes, per SWGA reaction. The addition of more target genomes improves the evenness of SWGA amplification, as well as the depth and breadth of coverage after sequencing.

Combing SWGA and MDD, Sundararaman and colleagues produced the first full-length genomes of *Plasmodium* species from naturally infected chimpanzees. Analyses of these sequences revealed that chimpanzee relatives of *P*. *falciparum* exhibit 10-fold higher within-species diversity than *P*. *falciparum*, suggesting that the jump from apes to humans may have occurred within the last 10,000 years [32,33]. Comparative genomics from SWGA-enriched samples also identified a horizontal transfer of 2 essential invasion genes, as well as the duplications and rapid diversification of the FIKK multigene family of protein kinases, which have been implicated in remodeling infected erythrocytes [32]. These adaptations may have provided the necessary tools for *Plasmodium* to transmit and adapt to humans [54].

Studies of African apes also revealed close genetic relatives of *P*. *vivax* in both chimpanzees and gorillas. Loy and colleagues used SWGA to produce near full-length genomes of 2 *P*. *vivax*-like parasites from chimpanzees [34]. These data showed that, like *P*. *falciparum*, *P*. *vivax* strains circulating in chimpanzees and gorillas in Africa are 10 times more diverse than their human counterparts, suggesting an African origin for this important human pathogen and an extreme bottleneck in human *P*. *vivax* [34]. This contradicted the previous belief that *P*. *vivax* originated as an Asian malaria [55].

### Challenges and opportunities for SWGA

**Fig 4:**
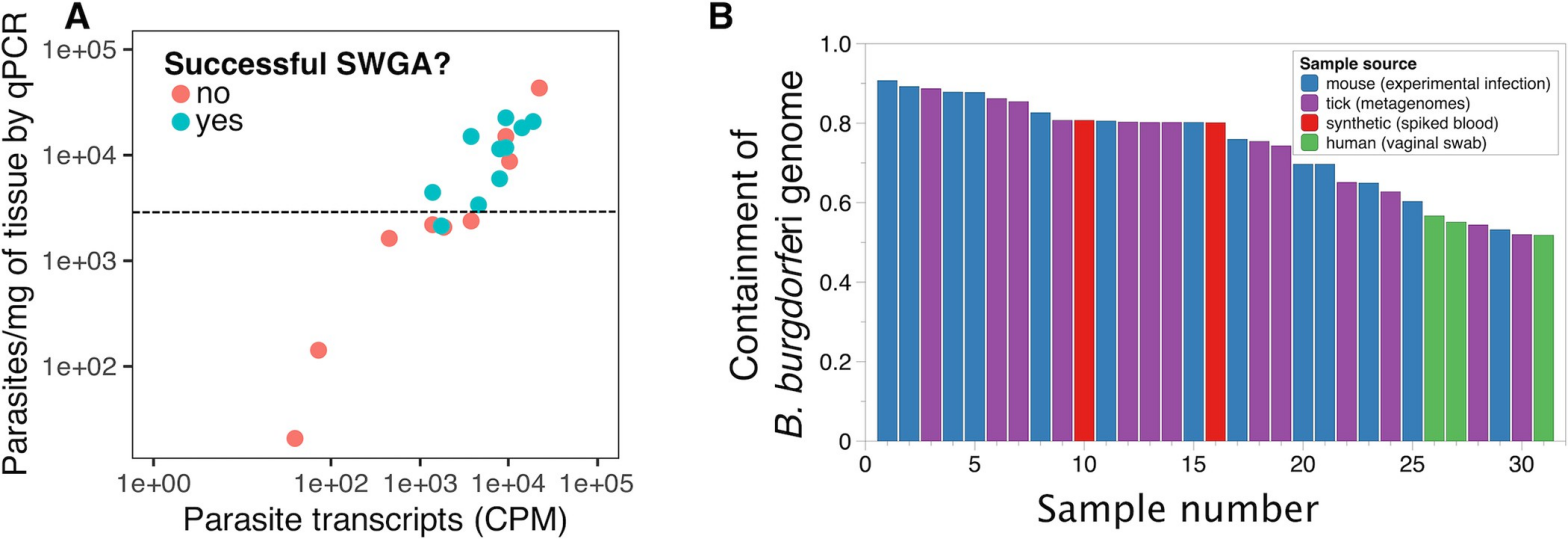
Overcoming limitations of low microbial load for SWGA. **(A)** Quantifying microbial load to prioritize samples for SWGA. Absolute quantification by qPCR (y-axis) of *L*. *braziliensis* from patient skin biopsies compared to relative quantification by RNA-seq (x-axis), from Pilling and colleagues [37]. Each point represents a single patient sample. Points are colored based on whether genomes were successful generated by SWGA for each patient. Dotted line indicates a potential qPCR quantification cutoff that could be used for prioritizing samples for SWGA. Below this threshold, SWGA failed for 6/7 samples but succeeded for 9/12 above. **(B)** Results from searching approximately 500,000 metagenomes from SRA using the *B*. *burgdorferi* strain B31 reference genome with Sourmash Branchwater software [65]. Each bar represents a sample colored by source.

SWGA fulfills an important need not currently met by traditional pathogen genomic approaches, such as amplicon tiling PCR for viral surveillance or WGS based on random hexamer priming. Despite the progression of SWGA from its initial development for bacteria to rapid adaptation for use in eukaryotic microbial genomics **(Fig 2)**, the latter has largely been limited to *Plasmodium* in blood samples **(Fig 3B and 3C)**, and only 2 neglected tropical diseases (filariasis [56] and leishmaniasis [37]) have been explored using this method. For SWGA to be more widely used across a diverse range of infectious diseases and sample types, there need to be improvements in method development. For example, we observed a relatively low success rate (approximately 25%) for SWGA on skin biopsies from patients harboring *L*. *braziliensis* infections [37], which can likely be attributed to the extremely low abundance of this pathogen together with the high levels of contaminating host DNA in solid tissue. This is supported by patient data showing that *L*. *braziliensis* burden is positively correlated with SWGA success **(Fig 4A)**. Work on viruses has shown that when pathogen burden is low, primer dimer interactions increase resulting in amplicon “drop out,” which can be improved by primer redesign [27]. Moreover, for reasons that are not well understood, SWGA primer sets that yield a genome from one sample may not be successful for other samples, necessitating the use of multiple primer sets for each sample assayed [21,37]. The consequence of these shortcomings is that many SWGA reactions need to be carried out in parallel to successfully generate genomes for a microbe of interest. Fortunately, newer methods for HTS library preparation dramatically reduce the cost of moving large numbers of SWGA reactions forward for sequencing [57]. One area for improvement would be to integrate artificial intelligence into the SWGA primer design software with experimental data—such as SWGA reaction success as assessed by quantitative PCR (qPCR) or coverage data from sequencing—to identify the reasons why some sets work well while others do not. Similar improvements have been described for viral surveillance to adapt PCR primers and pools as viruses evolve and mutate during an outbreak [27,58,59]. Improved SWGA primer set design would accelerate adoption of this method for insect vectors, fungi, and the microbiome, all of which are areas where population genomics is critical but where SWGA has not yet been applied.

One major difference between SWGA and traditional PCR is the up-front computational cost involved in primer set design. SWGA primer design requires working with at least 2 genomes (target microbe and host background) and searching a very large potential sequence space for short oligos that are likely to match better with the target microbial genome than the host background genome—a process that is computationally intensive [20,35]. This represents a potential impediment to wider adoption of the method, particularly by researchers in lower- and middle-income countries (LMICs) working on neglected tropical diseases, where SWGA could make a major contribution to population genomic studies of pathogens. Therefore, a major area for growth for SWGA would be to develop a resource analogous to the ARTIC network for real-time molecular epidemiology and outbreak responses for viruses. Such a resource could provide online tools for SWGA primer design (e.g., similar to PrimalScheme for viruses [22]) and standardized protocols for carrying out SWGA and analyzing the resulting data. The biology of parasitic helminths and protozoa presents an interesting challenge for SWGA primer design. These microbes frequently have life cycles that involve multiple evolutionarily divergent host species. Our own data suggest that primers designed using one mammalian host as background (e.g., human) can work well when performing SWGA in another mammalian host (e.g., mouse), but not in more distantly related species such as an insect vector [37]. Therefore, an ideal primer design resource would allow users to generate SWGA primer sets for different hosts, effectively allowing population genomics to be carried out across the complete life cycle for a parasite species.

Given the issues with low success rate described above, identifying samples with the greatest potential to yield complete or nearly complete genomes by SWGA is critical. qPCR for microbe-specific marker genes is one simple way to prioritize samples based on amount of target gene present [37] **(Fig 4A)**, but computational approaches may also help guide this prioritization process. Text matching algorithms, such as MinHash, have been adapted to search vast DNA sequence collections to identify samples that contain a microbial genome of interest [60–63], allowing rapid searching of nearly 1 million metagenomic samples publicly available through the Sequence Read Archive (SRA) [64,65] on a standard laptop computer [65]. Such large-scale in silico screens could be used to identify ideal samples, sample types, disease states, or experimental models that may yield the best results with SWGA. For example, searching all SRA metagenomes for *B*. *burgdorferi*, the cause of Lyme disease and a notoriously difficult pathogen to detect in vivo and culture in vitro, returns 31 samples from 3 public datasets (PRJNA208535, PRJNA723600, and PRJNA981116) that contain greater than 50% of the bacterial genome (inferred by shared k-mer content, or “containment”) **(Fig 4B)**. Not suprisingly, among these samples are total DNA extracts from homogenates of whole *Ixodes scapularis* (deer tick) **(Fig 4B, purple bars)**, as well as human blood spiked with *B*. *burgdorferi* DNA **(Fig 4B, red bars)** [66]. Nearly half of the samples identified in this screen were heart, skin, and joint tissue from experimentally infected mice **(Fig 4B, blue bars)** [66], while 3 samples were from vaginal swabs collected from a single human individual in a microbiome study of bacterial vaginosis [67]. Interestingly, *B*. *burgdorferi* has previously been isolated from vaginal secretions [68] and can cause genital ulcers [69–71]. Collectively, these data underscore how in silico screens can identify sample types and disease states potentially amenable to SWGA. Regardless of how samples are selected for SWGA, the primers do not bind exclusively to the target genome, thus the resulting sequence data will still contain a substantial fraction of reads from the background genome(s). These contaminating reads represent wasted sequencing effort and increase experiment costs. Two recent publications combined SWGA with adaptive sequencing [72] on the Oxford Nanopore Technologies platform for use in genome assembly and genomic surveillance of *Plasmodium* spp. [73–75], enabling real-time selective sequencing of only the target microbial species for as little as $25 per sample.

## Other methods for targeted enrichment of pathogen genomes

While this review primarily focuses on SWGA, other methods have been developed for the targeted enrichment of pathogens from complex samples. For example, hybrid capture sequencing is a widely used method that employs custom biotinylated oligonucleotide probes, or “baits,” to capture the target genome allowing unbound contaminating DNA to be washed away, thus enriching for the genome of interest. Although initially developed for the capture of the human exome [76], hybrid capture (e.g., Agilent SureSelect system) has been successfully adapted for many of the pathogens described above [39,43,77–83]. In contrast to SWGA, however, hybrid capture requires expensive baits and high amounts of input DNA (>100 ng), thus limiting its widespread use in LMICs and in situations where the input sample may be limiting [84]. While hybrid capture is a positive selection method, negative selection approaches can also achieve target enrichment through depletion of host DNA. For example, in *P*. *falciparum* studies, leukocyte depletion has been used to remove nucleated host cells [38,40], but this method is time consuming, requires refrigeration, and cannot be used when blood volume is low [38,46,47,85]. Alternatively, differential DNA methylation patterns between humans and microbes can be exploited to selectively digest host DNA in complex samples [47,85–88]. This method may offer one appealing solution for *Leishmania*, particularly since *L*. *donovani* reportedly lacks C-5 DNA methylation [89], opening the doors to using methylation-sensitive restriction enzymes to preferentially degrade host DNA. Restriction enzymes paired with SWGA could improve SWGA success rate for *L*. *braziliensis*. However, multiple methylation-sensitive restriction enzymes have been tested in *Plasmodium* SWGA studies with varying rates of success. Finally, although several commercial kits have been developed for host depletion [90], SWGA required 9-fold less genomic DNA copies/μl compared to some of these kits to generate the same quality of genome sequence for *T*. *pallidum* [30].

## Conclusions

Genome-wide studies of pathogens allow us to investigate population structure and transmission dynamics, link pathogen genotypes to pathogen virulence and persistence or host clinical traits, and monitor drug resistance genes in the population. SWGA complements amplicon tiling PCR for viral surveillance and WGS for generating microbial genomes from pure cultures. This method also extends the population genomics toolkit for pathogens by unlocking comparative genomics in less-than-ideal circumstances, including for pathogens that persist at extremely low abundance, are not experimentally tractable, or have reservoirs in endangered or protected host species such as gorillas and chimpanzees. In addition, eukaryotic pathogens not only have large and complex genomes [91], but they are also the cause of many neglected tropical diseases that lead to significant morbidity and mortality in LMICs. SWGA has and will continue to unlock genomic surveillance for this important group of organisms and, when combined with mobile and adaptive sequencing technologies, will help to decentralize the process of tracking local infection dynamics [92,93].
